# Enhancing Singlet Oxygen Generation in Conjugates of Silicon Nanocrystals and Organic Photosensitizers

**DOI:** 10.3389/fchem.2020.00567

**Published:** 2020-07-17

**Authors:** Deski Beri, Marius Jakoby, Dmitry Busko, Bryce S. Richards, Andrey Turshatov

**Affiliations:** ^1^Institute of Microstructure Technology, Karlsruhe Institute of Technology, Eggenstein-Leopoldshafen, Germany; ^2^Light Technology Institute, Karlsruhe Institute of Technology, Karlsruhe, Germany

**Keywords:** singlet oxygen, silicon nanocrystals, photosensitizers, NIR luminescence, microwave synthesis

## Abstract

Silicon nanocrystals (SiNCs) are regarded as a green and environmentally friendly material when compared with other semiconductor nanocrystals. Ultra-small SiNCs (with the size 4.6–5.2 nm) demonstrate strong UV absorption and photoluminescence in the near infrared (NIR) range with the high photoluminescence quantum yield (PLQY) up to 60%. In contrast to nanoporous silicon, ultra-small SiNCs do not possess an intrinsic ability to generate singlet oxygen (^1^O_2_). However, we demonstrate that SiNC-dye conjugates synthesized *via* microwave assistant hydrosilylation reaction produce ^1^O_2_ with moderate quantum yield (Φ_Δ_) up to 27% in cyclohexane. These interesting results were obtained *via* measurements of singlet oxygen phosphorescence at 1,270 nm. SiNCs play an important role in the production of singlet oxygen as SiNCs harvest UV and blue radiation and transfer absorbed energy to a triplet state of the attached dyes. It increases the population of the triplet states and leads to the enhancement of the singlet oxygen generation. Simultaneously, the SiNC-dye conjugates demonstrate NIR luminescence with the PLQY up to 22%. Thus, the luminescence behavior and photosensitizing properties of the SiNC-dye conjugates can attract interest as a new multifunctional platform in the field of bio-applications.

## Introduction

Singlet oxygen (^1^O_2_) is an extremely reactive species and powerful oxidant for many types of organic materials (Ogilby, 2010). The study of ^1^O_2_ has attracted increasing attention due to its potential applications in many fields such as chemical synthesis (Manfrin et al., 2019), photocatalysis (Nosaka and Nosaka, 2017), water purification (García-Fresnadillo, 2018), and photodynamic therapy of cancer (Wang et al., 2004; Vlaskin et al., 2009; Ghogare and Greer, 2016). In chemistry, ^1^O_2_ has been used to produced oxygenated hydrocarbons such as endoperoxide (Ahuja et al., 2018), deoxetanes (Camussi et al., 2019), as well as hydroperoxide and phosphine oxide for biomimetic organic synthesis of natural products and drugs (You and Nam, 2014). In the field of water treatment, ^1^O_2_ has demonstrated its efficiency in the degradation of water born pollutants (Lyubimenko et al., 2019). In photodynamic therapy, ^1^O_2_ has displayed a huge potential to destroy cancer cells (Campillo et al., 2019; Sun et al., in press). When a source of ^1^O_2_ is selectively delivered to a tumor affected tissue, ^1^O_2_ can react with many biological molecules—amino acid residues in proteins and the nucleobases in DNA and RNA resulting in photo induced degradation of cancer cells (Castano et al., 2004; Yang et al., 2019).

The most important conventional method to produce ^1^O_2_ is irradiation of photosensitizers (PSs) with ultraviolet or visible (UV/Vis) light. In the past, many potential organic and inorganic PSs have been proposed, for example, organic chromophores (Yogo et al., 2005), metal complexes (Monro et al., 2019), metal organic frameworks (Hu et al., 2018; Zheng et al., 2018), semiconductor quantum dots (QDs) (Bakalova et al., 2004; Rakovich et al., 2010), graphene QDs (Ge et al., 2014), perovskite nanocrystals (Gu et al., 2020) as well as metal nanoparticles (Chadwick et al., 2016) and metal nanowires (Smith et al., 2015). Among these, organic chromophores have been most intensively studied for ^1^O_2_ generation as they exhibit strong UV/Vis absorption, fast and efficient intersystem crossing (ISC), and a long triplet lifetime (Callaghan and Senge, 2018). When exposed to UV/Vis light, organic PSs produce a large number of long-lived triplet states, which transfer energy to the ground (triplet) molecular oxygen state *via* triplet energy transfer (Wang et al., 2004; Maisch et al., 2007). Despite the forbidden nature of ISC in quantum mechanics under the El-Sayed rule, this process can be partially allowed in organic systems if the ISC involves a change of the orbital type (Marian, 2012) or in systems with strong spin-vibronic coupling (Penfold et al., 2018). Heavy elements anchored to a chromophore can also significantly enhance the rate of population of the triplet state *via* spin–orbit effect (Marian, 2012). Recently, the population of triplet state *via* a SOCT (spin–orbit charge transfer)-ISC have been proved to be efficient for generation of ^1^O_2_ in different organic and water media (Filatov, 2020).

Interestingly, it has also been observed that the abundant and non-toxic chemical element of silicon (Si) can also produce ^1^O_2_. A pioneering work by Kovalev et al. (2002) described generation of ^1^O_2_ using silicon nanocrystals (SiNCs) distributed in a solid state porous Si layer. Several follow-up communications reported generation of ^1^O_2_ in organic and aqueous media by relatively large (with size 50–150 nm) porous SiNCs (Osminkina et al., 2011; Xiao et al., 2011). Photosensitization of ^1^O_2_ using ultra-small blue-emitting silicon nanocrystals (SiNCs) with size of 3 ± 1 nm and short photoluminescence (PL) decay lifetime of 1 ns was described by Llansola Portolés et al. (2010). Unlike SiNCs with blue PL resulting from surface defects, ^1^O_2_ generation with SiNCs demonstrating quantum confinement effect and optical properties similar to semiconductor QDs has not yet been reported. These SiNCs typically demonstrate strong UV absorption and bright long-lived PL (~100 μs PL decay time) in the red and near-infrared (NIR) spectral range. The long-lived NIR PL and low toxicity (Durnev et al., 2010; Cao et al., 2017; Mazzaro et al., 2017; Pramanik et al., 2018; Zhi et al., 2018) of SiNCs attract high attention of researchers in many application fields (Mazzaro et al., 2017).

The conjugation of organic dyes and SiNCs is a little explored topic relevant for many potential applications. It is known that an interaction between dyes and QDs can modified photophysical properties of the both (Lu et al., 2020). In particularly, the triplet exciton transfer between a dye and QD rises strong interest in the luminescent energy harvesting by singlet fission (Gray et al., 2020) and triplet fusion (Xia et al., 2020). Beside of that, QDs can enhance ISC in organic dyes anchored to their surface (Ahmed et al., 2015; Jin et al., 2019), that can be used for efficient generation of and ^1^O_2_ for PDT and photocatalytic applications.

Recently, we investigated conjugates of SiNCs and organic chromophores (Beri et al., 2020) in order to enhance visible absorption of SiNCs. To our surprise, we observed significant quenching SiNCs PL induced by organic chromophores covalently attached to the surface of SiNCs. We postulated that the quenching of the PL signal can originate from energy transfer from the SiNCs to the triplet state of the anchored dye molecule. These interesting findings motivated us to investigate in detail the role of SiNCs in photosensitization of the dye triplet state and a possible use of this process for generation of ^1^O_2_.

In the current paper, we anchored two different perylene derivatives to the surface of SiNCs using a thermal hydrosilylation reaction. The perylene unit in the close proximity to the surface of SiNCs plays the role of an energy acceptor mediating the energy transfer from SiNCs to the triplet state of molecular oxygen. Utilizing this mechanism of the ^1^O_2_ generation, we intended to enhance the potential value of SiNCs as a non-toxic and environmentally friendly material to sensitize reactive ^1^O_2_ for applications in chemical synthesis and photodynamic therapy.

## Materials and Methods

**Figure 1:**
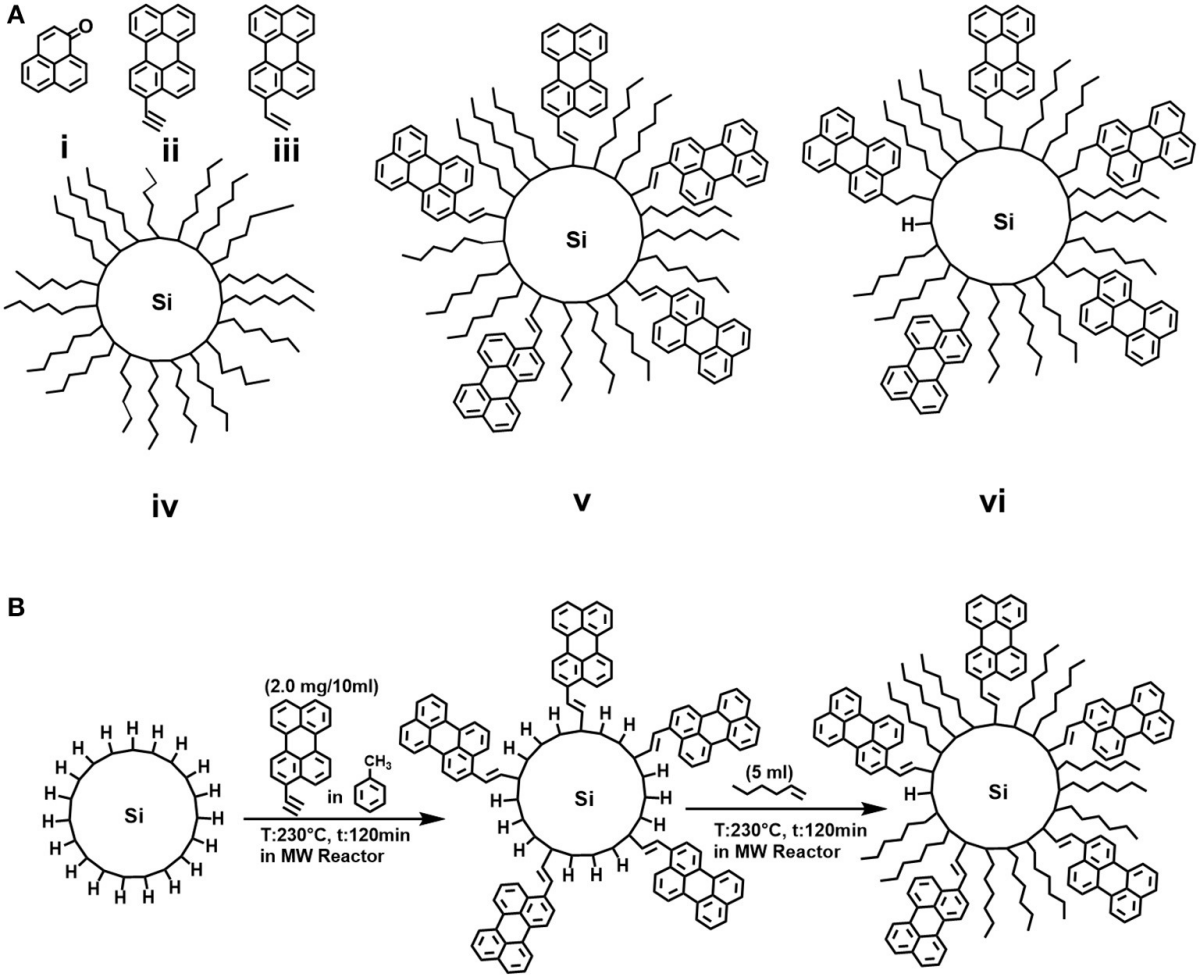
**(A)** Molecular structures of (i) phenalenone (*phe*), (ii) 3-ethynylperylene (*dye-1*), (iii) 3-ethenylperylene (*dye-2*), (iv) hexyl-functionalized SiNCs (*C6-SiNCs*), (v) 3-ethynylperylene/hexyl-functionalized SiNCs (*C6-1-SiNCs*) and (vi) 3-ethenylperylene/hexyl-functionalized SiNCs (*C6-2-SiNCs*); **(B)** Schematic representation of the hydrosilylation reaction of hydrogen-terminated SiNCs with dye and 1-hexene.

Silicon monoxide (99.9%, 325 mesh) and phenalenone (*phe*) (also known as perinaphtenone, 97%) were purchased from Sigma-Aldrich. Hydrofluoric acid (48%) was purchased from Fisher Scientific. Ethanol (98%), methanol (HPLC grade), toluene (99%+) were purchased from Merck. 1-hexene (C6) (99%) was purchased from Acros. Cyclohexane (spectroscopic grade) was purchased from Alfa Aesar. 3-ethynylperylene (*dye-1*) was purchased from Lumiprobe GmbH, Germany and 3-ethenylperylene (*dye-2*) was purchased from Fluorochem. Ltd, U.K. All chemicals were used without further purification and the chemical structure the most important substances are presented in Figure 1A.

### Synthetic Methods

Silicon nanocrystals were produced by a top-down method using the disproportionation reaction of silicon monoxide (SiO_x_), x < 1. The reaction initialized by annealing of 3.0 g SiO_x_ powder in a quartz boat at 900°C for 60 min under continuous H_2_/Ar (5:95%) flow. During this annealing process, the nucleation of SiNCs seeds inside a silicon dioxide matrix occurs (Hessel et al., 2012). Details regarding the annealing procedure can be found in the literature (Beri et al., 2018). After annealing, the sample was transferred to an agate mortar and ground.

#### Synthesis of Hydrogen Terminated SiNCs (H-SiNCs)

1.0 g the ground powder was transferred to a PTFE flask, followed by the addition of 10 ml absolute ethanol and stirring for 5 min. Subsequently, 10 ml of HF 48% was added to the flask and the solution was stirred for the next 2.5 h. In 50 ml PTFE separatory funnel, 15 mL of toluene was added followed by the addition of the ethanol/HF mixture. The separation of *H-SiNCs* was performed by extracting the non-polar (toluene) part from the polar (ethanol/HF) media. The toluene solution transferred to the centrifuge tube to remove the large particles. The centrifugation was performed at 2,000 rpm for 2 min. The big particles were discarded and the toluene solution was transferred to the G30 microwave (MW) tube (Anton Paar) and Ar was purged through the dispersion in order to remove dissolved O_2_. These *H-SiNCs* was ready for further reaction inside the MW reactor.

#### Synthesis of Hexyl Terminated SiNCs (C6-SiNCs)

Five milliliter of 1-hexene (C6) injected into G30 MW tube with the solution of *H-SiNCs* in toluene and purged for another 20 min. The tube with the solution was heated at 230°C for 120 min inside the MW reactor (Anton Paar GmbH). Unreacted C6 and toluene were removed with rotary evaporator and *C6-SiNCs* were re-dispersed in cyclohexane.

#### Synthesis of dye Terminated SiNCs (C6-1-SiNCs and C6-2-SiNCs)

A solution of *dye-1* in toluene (2 mg in 10 ml of toluene) was added to the solution of *H-SiNCs* in the G30 MW tube and the resulted solution was purged with Ar for 20 min. The tube with the solution was heated at 230°C for 120 min inside the MW reactor (Anton Paar GmbH). Five milliliter of *C6* was injected into the tube and again purged with Ar for another 20 min. The reaction in the MW reactor was repeated at 230°C for another 120 min. The schematic representative of the synthetic method is shown in Figure 1B. The product of the reaction with a dark orange color was transferred to the rotary evaporator flask and unreacted *C6* as well as toluene were removed. The obtained solid powder was rinsed with MeOH/EtOH (1:1) to remove unreacted dye. The precipitate was further redispersed in *cyclohexane* and stored in a glovebox and will be referred to as *C6-1-SiNCs*. The same procedure was applied for synthesis of *C6-2-SiNCs*.

### Sample Characterization

#### Size and Size Distribution

Transmission electron microscope (TEM) investigations were carried out on a TITAN 60–300 microscope at accelerating voltage 300 kV (Figure S1). Dynamic Light Scattering (DLS) using Anton Paar Litesizer 500 was used for characterization of the particle size distribution.

#### Absorption and Photoluminescence Excitation (PLE) Spectra

Absorption spectra were taken by UV-Vis-NIR spectrophotometer (Perkin Elmer Lambda 950). with a 2 nm resolution. Photoluminescence excitation (PLE) spectra were measured with a spectrofluorometer (Varian Cary Eclipse). The PLE scan was conducted monitoring the 800 nm emission (close to maximum peak of SiNCs emission) and exploring the excitation range from 300 to 550 nm.

#### Photoluminescence (PL) and Photoluminescence Quantum Yield (PLQY) Measurements

PLQY, PL-lifetimes and PL-emission were determined by the methods, which have been earlier described (Beri et al., 2020).

#### Singlet Oxygen Quantum Yields (Φ_Δ_)

Samples (2.5 ml) dispersed in cyclohexane were placed in a quartz cuvette (Starna) with path length 1 cm were irradiated with 405 nm diode lasers (75 mW, DL-7146-1012S, Roithner Laser Technique GmbH) or with a narrow-linewidth Ti:Sa laser (45 mW, SolsTiS, EMM-532, M-Squared Lasers) for the 317.5 nm excitations. The PL of ^1^O_2_ was measured with irradiance calibrated NIR spectrometer (NIRQuest 512-1.7, Ocean Optics) operating in 900–1,700 nm range. Integration time of 100 s was used for collection of the ^1^O_2_ phosphorescence spectra. The quantum yield of singlet ^1^O_2_ generation (Φ_Δ_) was calculated in agreement with Equation 1 using the *phe* as quantum yield as standard:

(1) $$\Phi_{\Delta }^{\text{x}}=\Phi_{\Delta }^{\text{R}}\frac{\left[{\text{S}}_{\text{em}}^{\text{x}}\right]}{\left[{\text{S}}_{\text{em}}^{\text{R}}\right]}\frac{\left[{\text{I}}_{\text{abs}}^{\text{R}}\right]}{\left[{\text{I}}_{\text{abs}}^{\text{x}}\right]}$$

where $\Phi_{\Delta }^{\text{x}}$ and $\Phi_{\Delta }^{\text{R}}$ are singlet oxygen quantum yields of the sample and the reference, respectively. $\left[{\text{S}}_{\text{e}\text{m}}^{\text{x}}\right]$ and $\left[{\text{S}}_{\text{e}\text{m}}^{\text{R}}\right]\;$are integrated area of ^1^O_2_ PL generated by the sample and the reference, while $\left[{\text{I}}_{\text{a}b\text{s}}^{\text{x}}\right]$ and $\left[{\text{I}}_{\text{a}b\text{s}}^{\text{R}}\right]$ are the number of absorbed photons by the sample and the reference. In case of the excitation with monochromatic light, absorption (% of absorbed light $\left[{\text{A}}_{\%}^{\text{x}}\right]$ and $\left[{\text{A}}_{\%}^{\text{R}}\right]$ at the excitation wavelength) of the sample and the reference can be used instead of $\left[{\text{I}}_{\text{a}b\text{s}}^{\text{x}}\right]$ and $\left[{\text{I}}_{\text{a}b\text{s}}^{\text{R}}\;\right]$.

The reported value of $\Phi_{\Delta }^{R}$ for *phe* in cyclohexane is 92 ± 10% (Schmidt et al., 1994). The concentrations of two sets of *phe, dye-1, dye-2, C6-1-SiNCs*, and *C6-2-SiNCs* solutions were adjusted to have roughly similar absorbance (*a*) at 405 and 317.5 nm.

#### Temperature-Dependent Photoluminescence Measurements

Dye molecules are dispersed in cyclohexane and placed in quartz cuvette with a path length of 2 mm and purged with argon gas for 30 min. Subsequently, the cuvette was clamped to the cryostat sample holder (Cryospares A7-103) and placed inside the sample chamber of the closed cycle cryostat (Oxford Instruments, Optistat Dry TLEX). After evacuating the sample chamber to ~10^−5^hPa, the chamber was flooded with helium (purity >99.999mol%) to improve thermal coupling between the sample and the heat exchanger of the cryostat. PL emission spectra were measured at 20K for both *dye-1* and *dye-2* samples. For the excitation, a mode-locked ytterbium laser (Light Conversion, Pharos) with a pulse width of 190 fs and a repetition rate of 20 kHz was used. The 1028 nm output of the laser was converted to 440 nm using an optical parametric amplifier (Light Conversion, Orpheus) and second harmonic generator (Light Conversion, Lyra). The steady state photoluminescence spectra were recorded by a fiber-coupled UV/VIS spectrometer (Avantes, AvaSpec-2048L).

## Result and Discussion

The molecular structures of the reference PS—*phe*, perylene derivatives *dye-1* and *dye-2*, as well as hexyl functionalized SiNCs (*C6-SiNCs*), hexyl-dye functionalized SiNCs (*C6-1-SiNCs* and *C6-2-SiNCs*) are shown in Figure 1A.

The unsaturated bonds of *dye*-1 and *dye-2* can react with the surface of *H-SiNCs* resulting in dye-functionalized SiNCs. The obtained dye-functionalized SiNCs exhibit enhanced absorption in the visible range and broad NIR emission with maximum of 860 nm and PLQYs of 15 ± 1% (for *C6-1-SiNCs*) and 22 ± 1% (for *C6-2-SiNCs*) as shown in Table 1. To improve the stability of SiNCs during and after the passivation reaction (Figure 1B), 1-hexene was employed as an additional surface ligand. A detailed investigation of the photophysical properties of *C6-SiNCs, C6- 1-SiNCs* and *C6-2-SiNCs* have been reported previously (Beri et al., 2020). In this previous publication, we found that the anchored dyes reduce both the PLQY of the NIR emission of SiNCs as well as the luminescence lifetime (at the NIR PL peak). As the NIR PL peak of SiNCs does not overlap with the absorption peak of the dyes (observed between 350 and 500 nm), we assumed that the NIR PL of SiNCs is quenched by the triplet state of the dyes *via* Dexter energy transfer. The resulting enhancement of the dye triplet population in the *C6-1-SiNCs* and *C6-2-SiNCs* can be probed *via* measurements of the yield of ^1^O_2_ generated by the triplet states of the dye. Thus, the main goal of the present study is to compare Φ_Δ_ under the direct excitation of the attached dyes (with 405 nm laser) and SiNCs (with 317.5 nm laser), with the two chosen wavelengths enabling this selectivity.

**Table 1:** Absorbance (α), normalized intensity of ^1^O_2_ phosphorescence (*I*_*em*_), photoluminescence quantum yield (*PLQY*), and singlet oxygen quantum yields (Φ_Δ_) measured with 317.5 nm and 405 nm lasers.

**PS**	**a(317.5nm)**	**I_em_^†^**	**a(405nm)**	**I_em_^‡^**	**PLQY,%**	****Φ_Δ_**,%^**†**^**	****Φ_Δ_**,%^**‡**^**
*phe*	0.576	1	0.760	1	–	92 ± 10*	92 ± 10*
*dye-1*	0.138	0.111	0.763	0.381	68 ± 1^‡, §^	45 ± 14	35 ± 5
*dye-2*	0.081	0.090	0.760	0.382	52 ± 1^‡, §^	56 ± 25	34 ± 5
*C6-SiNc*	0.780	–	0.773	–	33 ± 1^†, ♦^	–	–
*C6-1-SiNCs*	0.576	0.213	0.693	0.264	15 ± 1^†, ♦^	20 ± 5	27 ± 5
*C6-2-SiNCs*	0.331	0.052	n/a	n/a	22 ± 1^†, ♦^	9 ± 6	n/a

Corresponds to the 317.5 nm excitation;^‡^corresponds to the 405 nm excitation; *reference Φ_Δ_ = 0.92 ± 10 (Schmidt et al., 1994) ^§^PLQY of dyes visible emission integrated in the range 350–500 nm; ^♦^PLQY of SiNCs NIR emission integrated in the range 650–1,000 nm; I_em_ was normalized using the emission of ^1^O_2_ excited via phe (I_em_ = 1 for phe); The uncertainty of Φ_Δ_ was calculated in agreement with Equation S1 (Supporting Information). The uncertainty of PLQY measurements were earlier reported in Saleta Reig et al. (2020). The Φ_Δ_ for dye-2 under 405 excitation is not reported because luminescence of ^1^O_2_ was too weak.

There are two well-established methods to determine the Φ_Δ_. The first method uses a particular trap compound such as 9,10-dimethylanthracene (DMA), 1,3-diphenylisobenzofuran (DPBF), singlet oxygen sensor green, etc. (You, 2018). For instance, the DMA trap reacts specifically with ^1^O_2_ to form peroxide. This chemical reaction results in changes of the absorption spectrum of DMA decaying with irradiation time. By measuring the absorption decay, the Φ_Δ_ could be determined quantitatively *via* the comparison with the absorption decay induced by a reference PS with a known $\Phi_{\Delta }^{R}$. The second method is based on measurements of ^1^O_2_ phosphorescence and Equation 1. The radiative relaxation process from excited ^1^O_2_ to the ground triplet state (${}^{1}\Delta_{\text{g}}$→${}^{3}\Sigma_{\text{g}}$) yields an emission at 1,270 nm with relatively long lifetime (ms-to-s, depending on solvent) (Khan and Kasha, 1979; DeRosa and Crutchley, 2002). Similar to the first method, it also requires a reference material with known $\Phi_{\Delta }^{R}$ for the comparison of intensities of ^1^O_2_ phosphorescence.

It should be noted that the first method has a significant disadvantage for the estimation of Φ_Δ_. Several factors must be taken into account to determine the correct value of Φ_Δ_, including: overlap of absorption spectra of the sample and trap; self-degradation of the trap; as well as trap decomposition induced by other reactive oxygen species. For instance, we were not able to measure Φ_Δ_ *via* either DMA or DPBF due to the spectral overlap with the broad absorption spectra of *C6-1-SiNCs* and *C6-2-SiNCs*. A subsequent attempt to use rubrene (absorbing in range 450–550 nm) as the ^1^O_2_ trap also failed, as rubrene displayed a fast rate of the self-degradation upon irradiation with 405 nm and 317.5 nm lasers (Figure S2). Thus, the second method of the estimation of Φ_Δ_ based on the detection of ^1^O_2_ phosphorescence was chosen as the most reliable.

### Singlet Oxygen Generation With *Dye-1* and *Dye-2*

**Figure 2:**
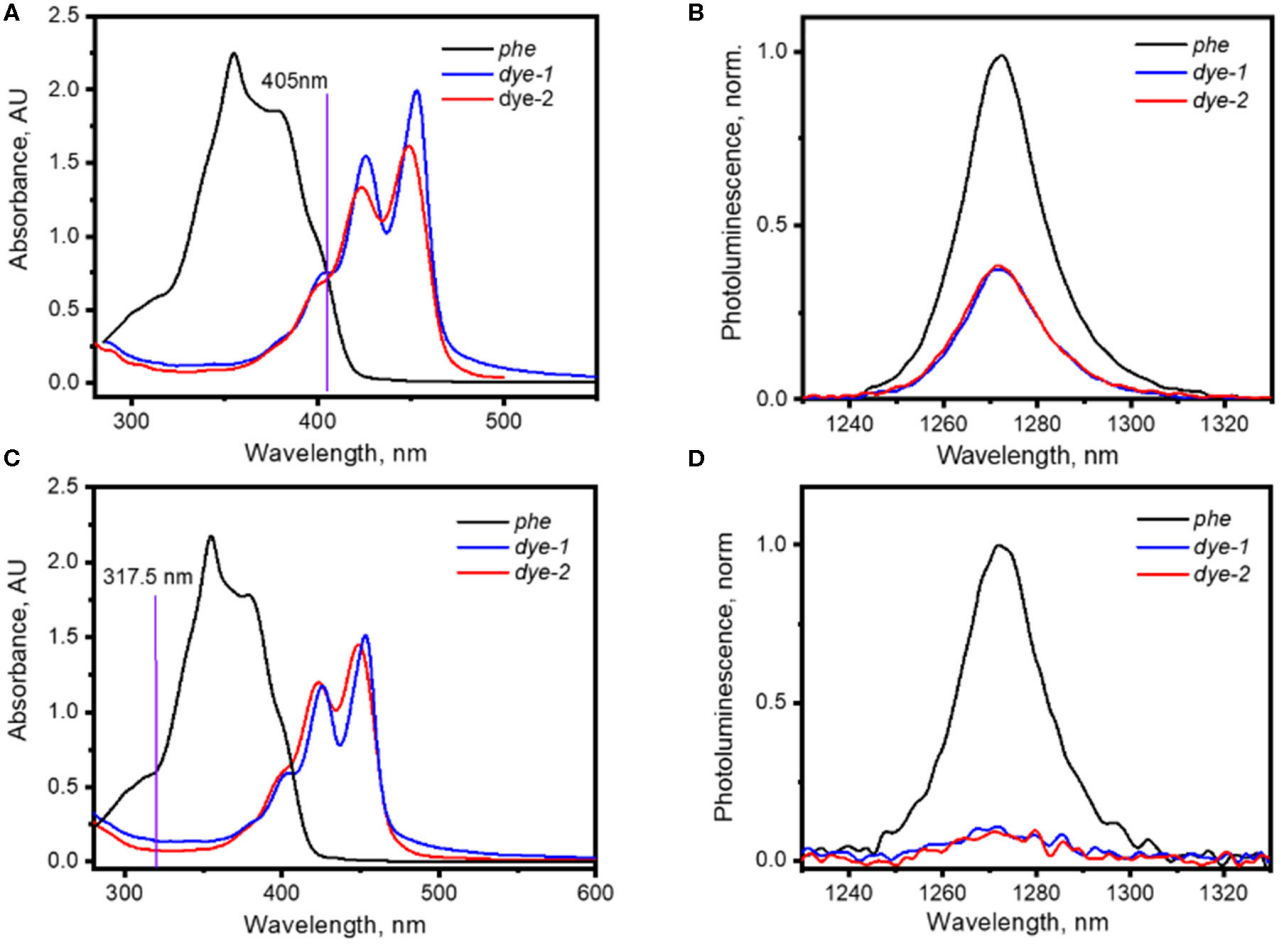
**(A)** UV-Vis absorption spectra of solutions of *phe, dye-1*, and *dye-2* in cyclohexane used for the generation of ^1^O_2_ with 405 nm laser; **(B)** ^1^O_2_ phosphorescence excited *via* irradiation of solutions of *phe* and *dye-1* with 405 nm laser (75 mW); **(C)** UV-Vis absorption spectra of solutions of *phe, dye-1*, and *dye-2* in cyclohexane used for the generation of ^1^O_2_ with 317.5 nm laser; **(D)** ^1^O_2_ phosphorescence excited via irradiation of solutions of *phe, dye-1*, and *dye-2* with 317.5 nm laser (excitation intensity of 15 mW).

Before conducting the experiments related to ^1^O_2_ generation, the photostability of the reference PS—*phe* was evaluated (Figure S3). The degradation of 20% *ph*e was found after long-time (1 h) irradiation of a solution of the reference PS in cyclohexane using 15 mW UV LED. Taking into account that acquisition of ^1^O_2_ luminescence (using 317.5 nm laser with intensity of 45 mW) takes ~100 s, we considered the reference PS as photostable. When we were satisfied that the *phe* was photostable, we investigated ^1^O_2_ generation by *dye-1* and *dye-2*. Perylene by itself exhibits a very high PLQY of 94% in cyclohexane (Taniguchi et al., 2018) and is thus a very poor PS. However, perylene derivatives have demonstrated ability to generate ^1^O_2_ with high quantum yield (Wu et al., 2010; Filatov et al., 2018; Blacha-Grzechnik et al., 2020). Figures 2B,D demonstrates phosphorescence of ^1^O_2_ generated *via* photoexcitation of *dye-1* and *dye-2* solutions in cyclohexane via both 405 and 317.5 nm laser excitation. The concentration of solutions with *dye-1, dye-2*, and *phe* was adjusted to have similar absorption at the excitation wavelengths (Figures 2A,C). The absorption of the samples $A_{\%}^{x}$ was estimated from absorbance *a*_*x*_ using Equation 2:

(2) $${\text{A}}_{\%}^{\text{x}}=100\%-10^{\left(2-{\text{a}}_{\text{x}}\right)},$$

where $A_{\%}^{x}$ is % of absorbed light and *a*_*x*_ is absorbance measured experimentally.

A calculation using Equation 1 gives the value of Φ_Δ_ = 35 ± 5% for *dye-1* and Φ_Δ_ = 34 ± 5% for *dye-2* when excited with the 405 nm laser. Measurements with the other excitation wavelength (317.5 nm) result to very weak ^1^O_2_ phosphorescence with very high uncertainty in Φ_Δ_ = 45 ± 14% (for *dye-*1) and Φ_Δ_ = 56 ± 25% (for *dye-2*) because of the weak dye absorption at 317.5 nm.

Interestingly, the dyes demonstrate unusually large values of Φ_Δ_ together with large values of absolute PLQY of 68 ± 1% and 52 ± 1% measured for *dye-1* and *dye-2*, respectively. Note, that absolute PLQYs were estimated in the integrating sphere and have a higher precision than Φ_Δ_. To gain inside ^1^O_2_ photosensitization we measured PLQY for dye solutions prepared inside a glovebox under oxygen-free conditions. We found PLQY of 98% for *dye-1* and 80% for *dye-2*. The obtained result indicates that photosensitization of ^1^O_2_ occurs solely *via* the excite singlet state in case of *dye-1* and predominantly in case of *dye-2*. It appears that, around two-thirds of the excited singlet states relax *via* the radiative transition for *dye-1*, whereas around one-third of the excited singlets transfer the energy to oxygen molecules. For *dye-2*, ~50% of the excitation energy decaying *via* radiative relaxation, whereas the remainder (~30%) transfers the energy to oxygen. Earlier, the highest Φ_Δ_ of 67% for perylene-like molecules was reported for rather complex di-(perylenebisimide) derivatives (Wu et al., 2010). However, our measurements indicate that moderate Φ_Δ_ of 35 ± 5% can be achieved with the simple molecules, which can be produced without expensive and time-consuming multistep synthesis.

**Figure 3:**
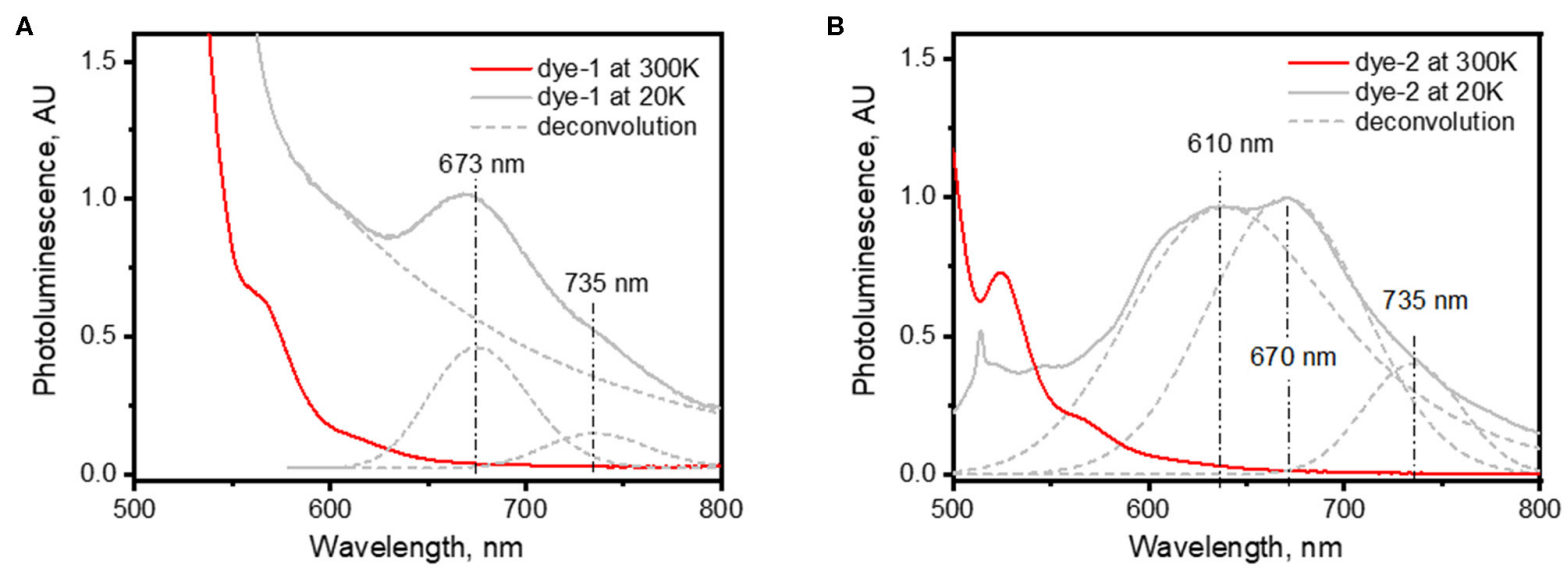
Photoluminescence of **(A)** *dye-1* and **(B)** *dye-2* at temperature of 300 and 20 K. Dashed lines represent results of deconvolution of low-temperature photoluminescence using Gaussian peaks centered at 673 and 735 nm for *dye 1* and 610, 670, and 735 nm for *dye 2*.

To obtain additional information about triplet states of *dye-1* and *dye-2*, we measured the emission spectra of their glassy solutions (in cyclohexane) at low temperature (20 K). Assuming small, but non-zero probability of ISC, we expected to detect phosphorescence of *dye-1* and *dye-2* at low temperature. Figure 3 demonstrates a comparison of PL spectra collected at room temperature and 20 K. Indeed, new emission bands appear in the low temperature spectra with maxima at 674 and 735 nm for *dye-1* and 670 and 735 nm for *dye-2*. We attributed the appearance of these bands to the radiative T_1_–S_0_ transition. The position of these peaks is slightly blue-shifted when compared with the position of T_1_ state of 800–850 nm in the unsubstituted perylene molecule (Turshatov et al., 2012). However, it is highly likely that the dye triplet with the T_1_ energy of ~1.7 eV (740 nm) can be excited *via* the energy transfer process utilizing the energy of SiNCs with PL in range 1.2–1.9 eV (650–1,000 nm).

### Singlet Oxygen Generation With *C6-1-SiNCs* and *C6-2-SiNCs*

The chemical reaction of the dyes with *H-SiNCs* yields a product of conjugation that demonstrates absorption of both components. The PLE spectra of *C6-1-SiNCs* (Figure S4) and *C6-2-SiNCs* (Figure S5) confirm the dye attachment. The excitation of SiNCs becomes possible *via* dye excitation in the range of 400–450 nm, which indicates the very short distance between dyes and SiNCs. In contrast, the physical mixture of *C6-SiNCs* and the dyes does not demonstrate NIR luminescence when the sample is excited with blue light (400–450 nm).

**Figure 4:**
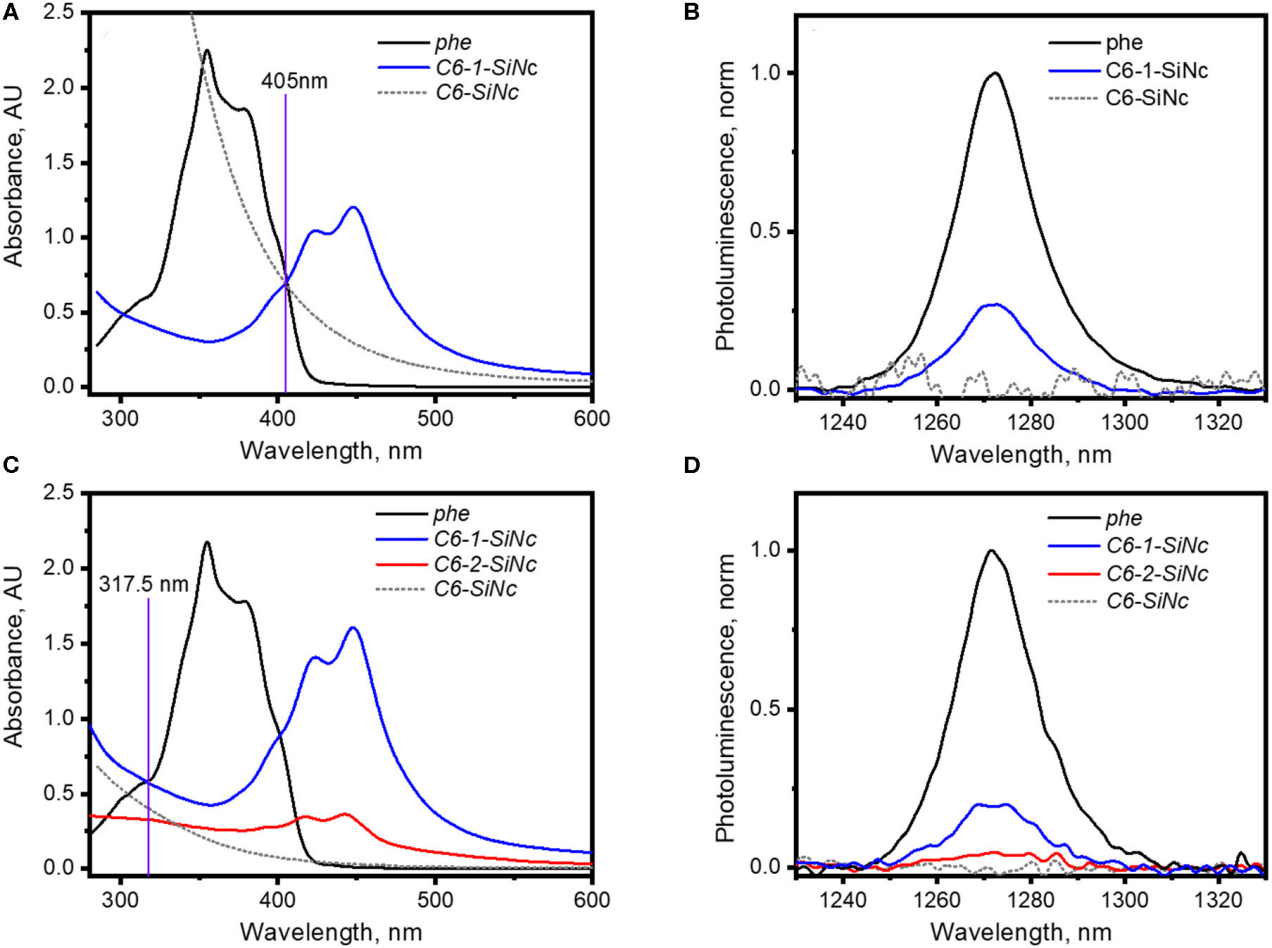
**(A)** UV-Vis absorption spectra of solutions of *phe, C6-SiNCs* and *C6-1-SiNCs* in cyclohexane used for the generation of ^1^O_2_ with 405 nm laser; **(B)** ^1^O_2_ phosphorescence excited *via* irradiation of solutions of *phe, C6-SiNCs* and *C6-1-SiNCs* with 405 nm laser (excitation intensity of 75 mW); **(C)** UV-Vis absorption spectra of solutions of *phe, C6-SiNCs, C6-1-SiNCs* and *C6-2-SiNCs* in cyclohexane used for the generation of ^1^O_2_ with 317.5 nm laser; **(D)** ^1^O_2_ phosphorescence excited via irradiation of solutions of *phe, C6-SiNCs, C6-1-SiNCs* and *C6-2-SiNCs* with 317.5 nm laser (excitation intensity of 45 mW).

It should be pointed out that the irradiation of *C6-1-SiNCs* and *C6-2-SiNCs* with 317.5 and 405 nm lasers excites different species. The 405 nm laser mainly excites the dye molecule anchored to the surface of SiNCs, whereas the 317.5 nm laser directly excites SiNCs as the dyes exhibit an absorption minimum at this wavelength. The results of the calculation with Equation 1 (using the data presented in Figures 4A,B and Table 1) indicate that *C6-1-SiNCs* excited with 405 nm laser generate ^1^O_2_ with Φ_Δ_ = 27 ± 5%. This quantum yield is lower than Φ_Δ_ of pure *dye-1*. However, the experiment emphasizes that the *C6-1-SiNCs* conjugate exhibits synergistic behavior. Under blue light excitation at room temperature, the nanoparticles demonstrate NIR emission (originating from the SiNCs core) with PLQY of 15 ± 1% and ^1^O_2_ generation (originated from the anchored dye). Thus, this new conjugate can attract potential interest in photomedicine as a new chemical agent combining properties of PS and a NIR phosphor.

The irradiation of *C6-1-SiNCs* with the 317.5 nm laser should lead to selective excitation of SiNCs. To the best of our knowledge, the SiNCs synthesized from SiO_x_ are not able to generate ^1^O_2_. Indeed, the excitation of *C6-SiNCs* with 317.5 and 405 nm lasers do not produce ^1^O_2_ phosphorescence (Figures 4B,D). However, the excitation of the *C6-1-SiNCs* conjugate with the 317.5 nm laser results in ^1^O_2_ phosphorescence with Φ_Δ_ = 20 ± 5%. The enhancement factor (F) of ^1^O_2_ oxygen generation with UV light (317.5 nm) for *C6-1-SiNCs* vs. *dye-1* can be determined using Equation 3:

(3) $$\text{F}=\frac{\left[\Phi_{\Delta }^{\left(\text{C}6\right)-1-\text{SiNCs}}\right]}{\left[\Phi_{\Delta }^{\text{dye}}\right]}\frac{\left[{\text{a}}_{\left(\text{C}6\right)-1-\text{SiNCs}}\right]}{\left[{\text{a}}_{\text{dye}}\right]}$$

where we assume that two solutions (*C6-1-SiNCs* and *dye-1)* exhibit similar absorbance at the 450 nm peak (indication of similar perylene concentration); $\Phi_{\Delta }^{\left(C6\right)-1-SiNCs}$ and $\Phi_{\Delta }^{dye}$ are quantum yields of ^1^O_2_ generation measured at the 317.5 nm excitation for the *C6-1-SiNCs* conjugate and *dye-1*, respectively; *a*_(*C*6)−1−*SiNCs*_ and *a*_*dye*_ are absorbance of the two solutions at 317.5 nm.

The enhancement factor *F* = 2.3 indicates that the solution with *C6-1-SiNCs* is able produce 2.3 times more ^1^O_2_ then the solution with *dye-1* with similar concentration of perylene chromophore. We performed here the calculation of *F* only for one single wavelength (317.5 nm). However, this conclusion can be also valid for the broad UV range (~300–350 nm) with strong absorption of SiNCs. Thus, the *C6-1-SiNCs* conjugate demonstrate the ability of efficient ^1^O_2_ generation over very broad spectral range utilizing the absorption of SiNCs (~300–350 nm) and the absorption of dye-1 (~350–460 nm).

It has been mentioned in our previous publication (Beri et al., 2020) that energy transfer from SiNCs to the triplet state of *dye-2* is less efficient. This observation was also confirmed in the experiment with ^1^O_2_ generation. The *C6-2-SiNCs* conjugate exhibits significant lower Φ_Δ_ = 9 ± 6% under excitation with UV light (317.5 nm) (Figures 4C,D and Table 1).

### Energy Transfer From SiNCs to Perylene Chromophore

**Figure 5:**
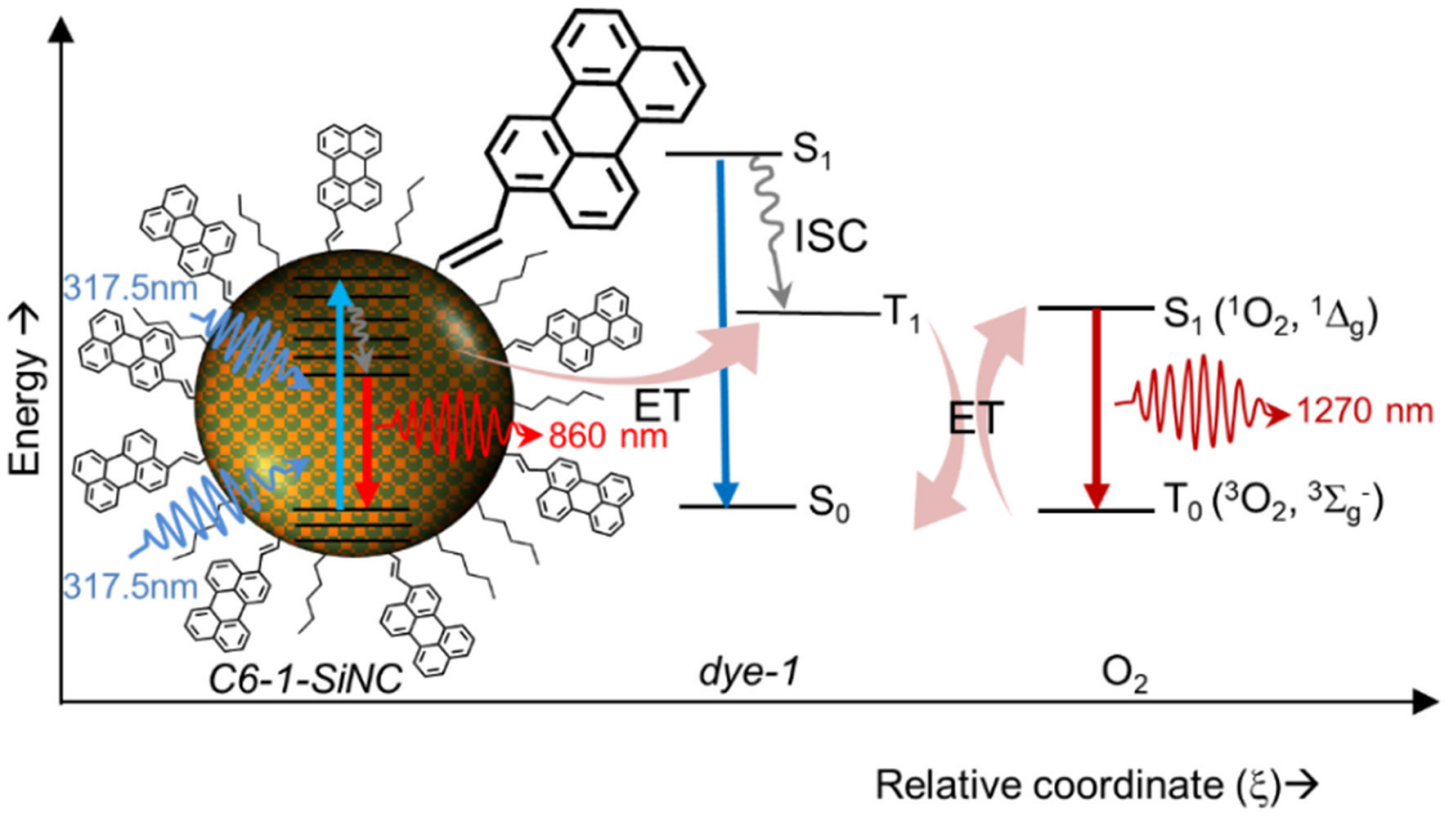
Schematic showing photosensitization of ^1^O_2_ with SiNCs using the attached dye as transmitter for the excitation energy.

The schematic at Figure 5 displays a ^1^O_2_ generation pathway under UV excitation of *C6-1-SiNCs*. Under excitation with 317.5 nm laser, the crystals emit NIR photons with the wavelength of 860 nm. At the same time, the excitation energy can be transferred to the triplet state of the dye. The triplet state interacts with molecular oxygen. The interaction produces ^1^O_2_ that emits NIR photons with the wavelength of 1,270 nm with the overall quantum efficiency of ^1^O_2_ production around 20%

Finally, we were able to evaluate the efficiency of energy transfer (η_*ET*_) from the SiNC core to the perylene chromophore from the calculations of Φ_Δ_. At 317.5 nm excitation, the efficiency of the energy transfer can be calculated using Equation 4 and the values contained in Table 1:

(4) $$\eta_{\text{ET}}=\frac{\left[\Phi_{\Delta }^{317.5\text{nm}}\right]-\left[{\text{P}}_{\text{dye}}^{317.5\text{nm}}\right]\left[\Phi_{\Delta }^{405\text{nm}}\right]}{\left[{\text{P}}_{\text{SiNCs}}^{317.5\text{nm}}\right]\left[\Phi_{\Delta }^{405\text{nm}}\right]},$$

where η_*ET*_ is energy transfer efficiency from SiNCs to attached dyes, $\left[\Phi_{\Delta }^{317.5nm}\right]=$ 20% is the quantum yield of ^1^O_2_ generation by *C6-1-SiNCs* under 317.5 nm excitation; $\left[\Phi_{\Delta }^{405nm}\right]=$ 27% is the quantum yield of ^1^O_2_ generation by *C6-1-SiNCs* under 405 nm excitation; $\left[P_{dye}^{317.5nm}\right]=$ 24% is a part of the excitation light (with wavelength of 317.5 nm) absorbed by the dye; $\left[P_{SiNCs}^{317.5nm}\right]=$ 76% is a part of the excitation light (with wavelength of 317.5 nm) absorbed by the SiNC core.

The calculation of η_*ET*_ with Equation 4 gives a value of 66%. This value is in very good agreement with the value of η_*ET*_ of 55% calculated using PL lifetimes of the NIR emission of *C6-SiNCs* and *C6-1-SiNCs* nanocrystals (Beri et al., 2020).

## Conclusions

The SiNCs were modified with organic dyes *via* the hydrosilylation reaction in the microwave reactor. The SiNC-dye conjugates were investigated for the first time within the context of singlet oxygen generation. The singlet oxygen yield was determined *via* measurements of singlet oxygen phosphorescence (at 1,270 nm) in cyclohexane solutions using the comparison with the singlet oxygen phosphorescence produced by the reference PS—*phe*. The Φ_Δ_ values were estimated for two excitation wavelengths: 317.5 nm at 405 nm. The calculation of Φ_Δ_ for the *C6- 1- SiNC* conjugate results Φ_Δ_ = 27 ± 5% and Φ_Δ_ = 20 ± 5% for 405 nm and 317.5 nm excitations, respectively. We attributed high yield of singlet oxygen generation under 317.5 nm with efficient energy transfer from photoexcited SiNCs to the triplet states of attached molecules of *dye-1*. In contrast to *dye-1, dye-2* is a less efficient acceptor for SiNCs. As results, the Φ_Δ_ value of the *C6-2-SiNCs* conjugates is smaller—Φ_Δ_ = 9 ± 6%. We assumed that *C6-1-SiNCs* demonstrate high Φ_Δ_ over entire absorption spectrum of *C6-1-SiNCs* (~300–460 nm). Thus, this finding indicates a large potential of the dye modified SiNCs for the production of singlet oxygen.

## Data Availability Statement

All datasets generated for this study are included in the article/Supplementary Material.

## Author Contributions

DBe: synthesis of the dye functionalized SiNCs, characterization of SiNCs, measurement of singlet oxygen, evaluation and interpretation of the data, and writing. MJ: temperature dependent photoluminescence measurement. DBu: measurement of singlet oxygen. BR: supervision, data interpretation, and writing. AT: development of a paper concept, supervision data interpretation, and writing. All authors contributed to the manuscript revision, read, and approved the submitted version.

## Conflict of Interest

The authors declare that the research was conducted in the absence of any commercial or financial relationships that could be construed as a potential conflict of interest.
