# Supplementary material for: Enhancing Singlet Oxygen Generation in Conjugates of Silicon Nanocrystals and Organic Photosensitizers
Source: Front Chem. 2020 Jul 17;8:567. doi: 10.3389/fchem.2020.00567 (PMC7379910; doi:10.3389/fchem.2020.00567)
Supplement: Supplementary file 1 [file Table_1.DOCX]

# Supporting Information

Enhancing singlet oxygen generation in conjugates of silicon nanocrystals and organic photosensitizers

Deski Beri, Marius Jakoby, Dmitry Busko, Bryce S. Richards,* Andrey Turshatov*

Transmission electron microscopy (TEM)


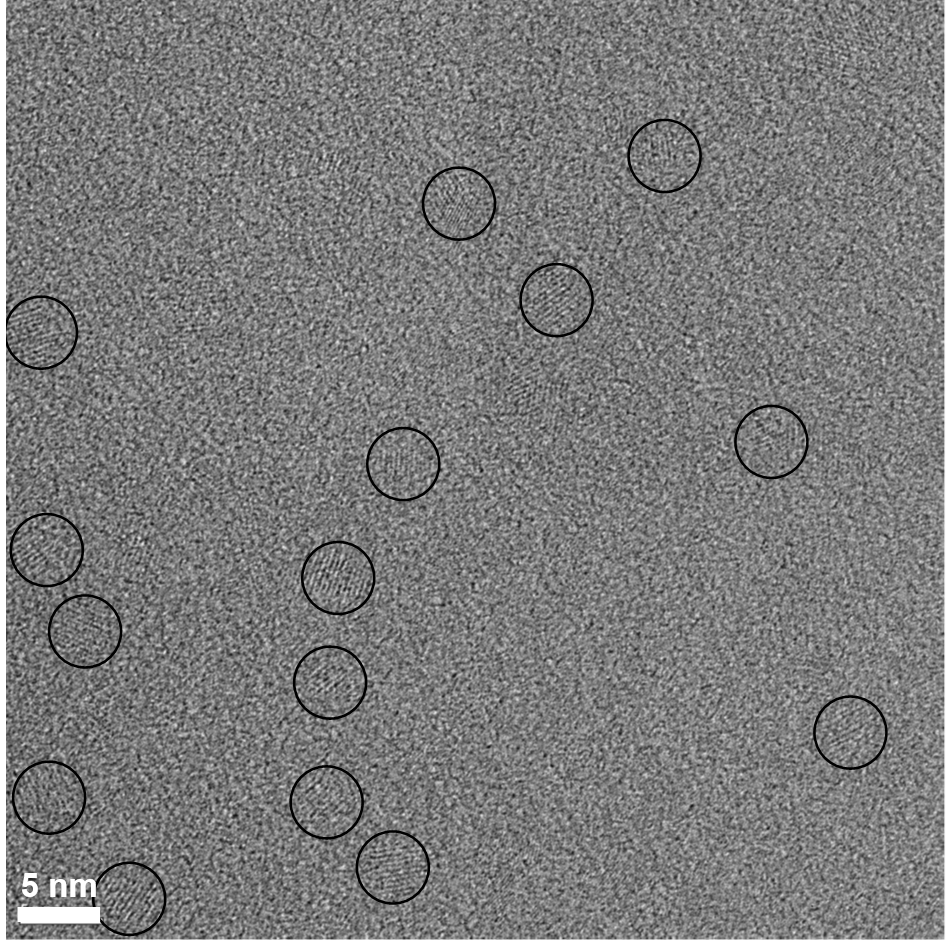


**Figure S1.** High resolution TEM image of C6-2-SiNCs. The TEM image taken at high magnification reveals the crystalline structure of nanoparticles, however accurate analysis of the SiNC size cannot be performed due to the poor contrast originated from low atomic weight of silicon

Photodegradation of rubrene

**
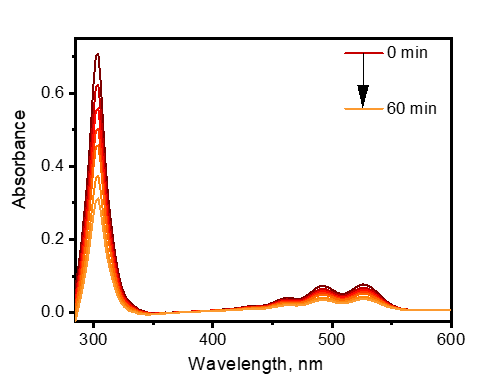
**

# Figure S2. Absorption spectra of solution of rubrene in cyclohexane monitored during 60 minutes of irradiation with 300 nm LED (with intensity of 15 mW).

Photodegradation of the reference photosensitizer


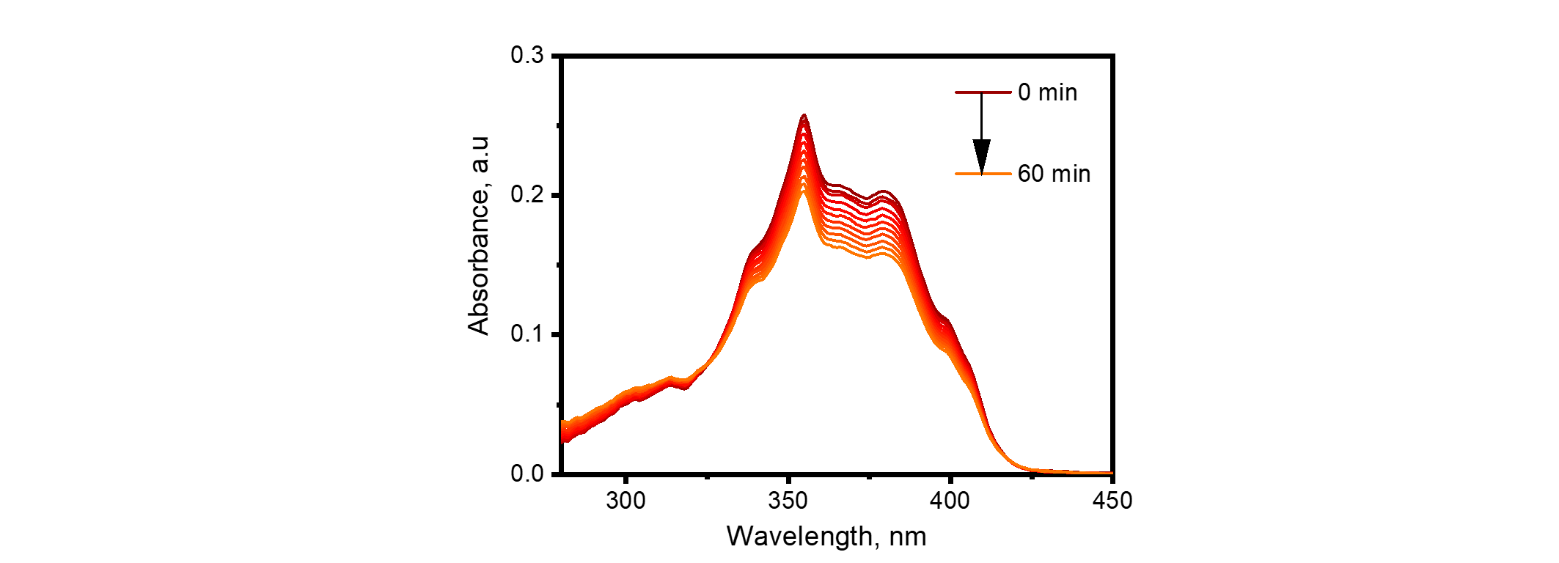


# Figure S3. Absorption spectra of solution of *phe* in cyclohexane monitored during 60 minutes of irradiation with 300 nm LED (with intensity of 15 mW).

PL and PLE spectra of *C6-1-SiNCs* conjugate


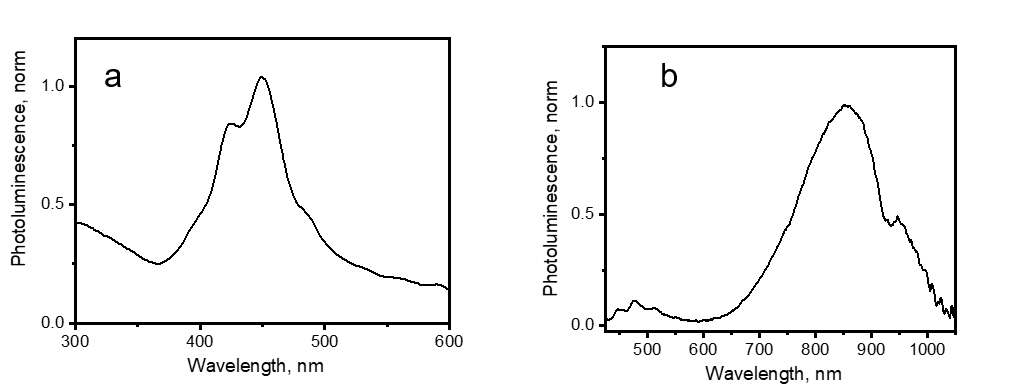
Figure S4. (a) Photoluminescence excitation spectrum of *C6-1-SiNCs* conjugate. The intensity of photoluminescence at 800 nm was measured as function of the excitation wavelength. The results indicate that NIR luminescence of *SiNCs* can be excited via excitation of *dye-1*. (b) Photoluminescence spectrum of *C6-1-SiNCs* excited at 405 nm. The spectrum consists of weak luminescence of *dye-1* (420-500 nm) and strong luminescence of *SiNCs* (650 – 1000 nm)

PL and PLE spectra of *C6-2-SiNCs* conjugate


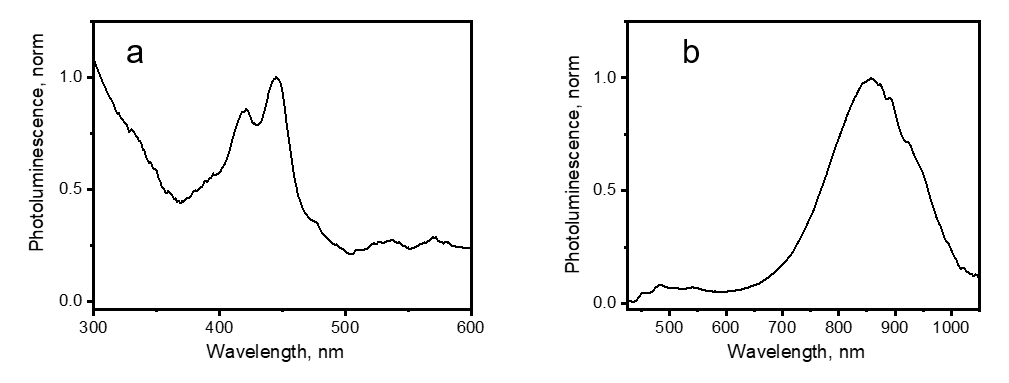
Figure S5. (a) Photoluminescence excitation spectrum of *C6-2-SiNCs* conjugate. The intensity of photoluminescence at 800 nm was measured as function of the excitation wavelength. The results indicate that NIR luminescence of *SiNCs* can be excited via excitation of *dye-2*. (b) Photoluminescence spectrum of *C6-2-SiNCs* excited at 405 nm. The spectrum consists of weak luminescence of *dye-2* (420-500 nm) and strong luminescence of *SiNCs* (650 – 1000 nm)

**Uncertainty of SO quantum yield**

The uncertainty of ΔΦ_Δ_ comprised the uncertainties from the following experimental steps:

- Δ$\Phi_{\Delta}^{R}$ = 0.5 % from the value of SO quantum yield of the reference PS
- Possible photobleaching of the reference PS ($\Delta S$ = 12 %)
- UV/Vis measurements ($\Delta UV/Vis$ = 0.1%)
- Luminescence detection ($\Delta Det$ = from 1% for strong luminescence in Figure 1b up to 19 % for weak luminescence in Figure 2d)

Thus, ΔΦ_Δ_ was calculated using Equation S1

$\Delta\Phi_{\Delta}=\sqrt{\left[ \Delta\Phi_{\Delta}^{R} \right]^{2}+\left[ \Delta S \right]^{2}+\left[ \Delta UV/Vis \right]^{2}+\left[ \Delta Det \right]^{2}}$ , (S1)

The results of calculations are presented in Table 1.
